# Solar Water Splitting with a Hydrogenase Integrated in Photoelectrochemical Tandem Cells

**DOI:** 10.1002/anie.201805027

**Published:** 2018-07-17

**Authors:** Dong Heon Nam, Jenny Z. Zhang, Virgil Andrei, Nikolay Kornienko, Nina Heidary, Andreas Wagner, Kenichi Nakanishi, Katarzyna P. Sokol, Barnaby Slater, Ingo Zebger, Stephan Hofmann, Juan C. Fontecilla‐Camps, Chan Beum Park, Erwin Reisner

**Affiliations:** ^1^ Department of Chemistry University of Cambridge Cambridge CB2 1EW UK; ^2^ Department of Engineering University of Cambridge Cambridge CB3 0FA UK; ^3^ Max Volmer Laboratorium für Biophysikalische Chemie, Sekretariat PC14 Institut für Chemie Technische Universität Berlin Straße des 17. Juni 135 10623 Berlin Germany; ^4^ Metalloproteins Unit Institut de Biologie Structurale Université Grenoble Alpes CEA, CNRS 38044 Grenoble France; ^5^ Department of Materials Science and Engineering Korea Advanced Institute of Science and Technology 291 Daehak-ro Yuseong-gu Daejeon 34141 Republic of Korea

**Keywords:** hydrogenase, photoelectrochemistry, photosynthesis, silicon, water splitting

## Abstract

Hydrogenases (H_2_ases) are benchmark electrocatalysts for H_2_ production, both in biology and (photo)catalysis in vitro. We report the tailoring of a p‐type Si photocathode for optimal loading and wiring of H_2_ase through the introduction of a hierarchical inverse opal (IO) TiO_2_ interlayer. This proton‐reducing Si|IO‐TiO_2_|H_2_ase photocathode is capable of driving overall water splitting in combination with a photoanode. We demonstrate unassisted (bias‐free) water splitting by wiring Si|IO‐TiO_2_|H_2_ase to a modified BiVO_4_ photoanode in a photoelectrochemical (PEC) cell during several hours of irradiation. Connecting the Si|IO‐TiO_2_|H_2_ase to a photosystem II (PSII) photoanode provides proof of concept for an engineered Z‐scheme that replaces the non‐complementary, natural light absorber photosystem I with a complementary abiotic silicon photocathode.

The capture and storage of solar energy in the form of H_2_ through water splitting is a promising process to produce sustainable fuel. Hydrogenases (H_2_ases) are metalloenzymes that operate at the thermodynamic potential for proton reduction, which makes them attractive noble‐metal‐free model catalysts.^1^ H_2_ases have been combined with a series of light absorbers, such as dye‐sensitized TiO_2_, carbon nitrides, cadmium‐based and carbon nanodots, In_2_S_3_ nanoparticles, and organic dyes, for photocatalytic H_2_ production in the presence of a sacrificial electron donor.^2^ Sacrificial reagents can be avoided by using a photoelectrochemistry (PEC) approach with H_2_ases wired to electrodes, but these systems have relied on an external applied voltage to drive water splitting into H_2_ and O_2_.^3^ Thus unassisted solar water splitting with a H_2_ase in vitro has been a long‐standing goal.

Silicon (Si) has a narrow band gap of 1.1 eV and is widely used as an efficient photocathode for proton reduction. Its use requires protection of the Si surface from the aqueous electrolyte solution (typically with a TiO_2_ coating) and modification with a H_2_ evolution catalyst.^4^ Previous reports on p‐Si photocathodes modified with H_2_ase suffered from low photocurrents or Faradaic efficiencies, which can be attributed to a small effective surface area of the electrode and suboptimal integration of the H_2_ase into the materials architecture.3c, 5

Herein, we report the assembly of a Si‐based photoelectrode that features a hierarchically structured inverse opal (IO)‐TiO_2_ layer optimized for high and stable integration of a [NiFeSe]‐H_2_ase from *Desulfomicrobium baculatum* as the H_2_ evolution biocatalyst.1b, 6 The Si|IO‐TiO_2_|H_2_ase photocathode can be coupled to complementary photoanodes for water oxidation to achieve overall water splitting (see the Supporting Information, Figure S1). We investigated coupling of the Si|IO‐TiO_2_|H_2_ase to an abiotic (n‐type BiVO_4_) and a biotic (Photosystem II, PSII) photoanodic system for overall water splitting.

A 4 nm thick TiO_2_ layer was deposited on the surface of a p‐Si wafer by atomic layer deposition (ALD) immediately after hydrofluoric acid (HF) treatment to protect the electrode from the formation of an insulating silica layer (Figures S2 and S3). A hierarchically structured IO‐TiO_2_ layer of 10 μm film thickness was subsequently assembled on top of the ALD layer by co‐assembly of TiO_2_ nanoparticles (P25, 21 nm) with polystyrene beads (750 nm), followed by heating at 450 °C.3a Characterization by scanning electron microscopy (SEM; Figures 1 A and S4) showed a macropore diameter of 750 nm, facilitating the penetration of large biomolecules. X‐ray diffraction (XRD) and UV/Vis spectroscopy confirmed the expected crystallinity and transparency in the visible spectrum for IO‐TiO_2_ (Figure S5).

**Figure 1:**
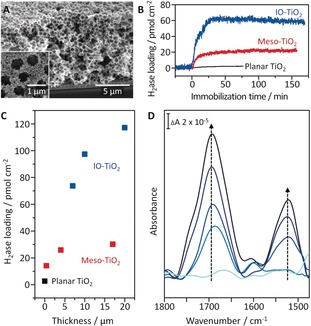
A) Cross‐sectional SEM image of the Si|IO‐TiO_2_ photocathode. Inset: Top‐view SEM. B) Loading capacities and stabilities of immobilized H_2_ase on planar, mesoporous (4 μm), and IO‐TiO_2_ (7 μm) electrodes studied by QCM analysis. C) QCM quantification of the H_2_ase loading on different TiO_2_ architectures with various film thicknesses. D) ATR‐IR spectra of Si prism|IO‐TiO_2_|H_2_ase during incubation with H_2_ase (10 μL of 8 μm) after 0, 7.5, 15, 22.5, and 30 min. The intensities of the amide I (1690 cm^−1^) and II (1520 cm^−1^) bands from the protein backbone of the H_2_ase molecules increased with time in direction of the arrows. The penetration depth of the evanescent wave into the bottom of the 10 μm thick IO‐TiO_2_ from the ATR‐Si prism surface is approximately 0.5 μm.

The ability of IO‐TiO_2_ to support high protein loadings was studied by quartz crystal microbalance (QCM) analysis. The IO‐TiO_2_ electrode at 7 μm thickness exhibited a 3 and 27 times higher loading capacity for H_2_ase than mesoporous (>4 μm thickness; Figure S6) and planar TiO_2_ electrodes, respectively (Figure 1 B). The protein remained almost quantitatively adsorbed on the porous TiO_2_ layers for more than two hours during the QCM measurement. The loading capacity of H_2_ase increased with the film thickness of the IO‐TiO_2_ layer, whereas the loading on the mesoporous TiO_2_ film saturated at a thickness of 4 μm (Figures 1 C and S6).

Penetration of the H_2_ase through the IO‐TiO_2_ architecture was then probed by attenuated total reflection infrared (ATR‐IR) spectroscopy using a Si prism coated with an IO‐TiO_2_ layer (10 μm thickness). After addition of H_2_ase (10 μL of 8 μm) to the buffer solution covering the IO‐TiO_2_ coated prism, two characteristic bands at 1690 cm^−1^ and 1520 cm^−1^, known as amide I (preferentially CO stretching) and amide II (mainly a combination of NH bending and CN stretching vibrations), were detected (Figure 1 D).^7^ The protein adsorption was monitored in situ and was still increasing after 30 min of incubation time. In this experimental setup, the penetration depth of the evanescent wave of the IR beam was restricted to approximately 0.5 μm from the Si prism surface, and the amide bands were therefore assigned to H_2_ase that had infiltrated the entire IO‐TiO_2_ layer. For comparison, ATR‐IR spectra of 1 μm thick mesoporous TiO_2_ on a Si prism exhibited no amide bands even after incubation with H_2_ase for 45 min (Figure S7). The hierarchical electrode structure has therefore been established as a superior scaffold for enzyme integration compared to meso‐ and flat TiO_2_.^5^ Thus, this Si|IO‐TiO_2_|H_2_ase was employed in all PEC experiments.

Drop‐casting of H_2_ase (80 pmol) onto the IO‐TiO_2_ layer was optimized by protein film voltammetry on FTO|IO‐TiO_2_|H_2_ase electrodes (FTO=fluorine‐doped tin oxide; Figure S8). The performance of Si|IO‐TiO_2_|H_2_ase as a photocathode was studied by linear sweep voltammetry (LSV) under chopped, UV‐, and IR‐filtered simulated solar light irradiation (100 mW cm^−2^; AM1.5G; *λ*>420 nm; 25 °C). The electrolyte solution (pH 6.0) for LSV contained 50 mm MES (2‐(*N*‐morpholino)ethanesulfonic acid) and 50 mm KCl. A photocurrent onset potential was observed at approximately 0.35 V vs. the reversible hydrogen electrode (RHE), which is only slightly more positive than in H_2_ase‐free Si|IO‐TiO_2_ (Figures 2 A and S9 A). The photocurrent onset potential is therefore predominantly controlled by the Si‐TiO_2_ interface,^8^ and the photocurrents on the short timescale of a voltammetric scan contain a significant contribution from the charging process of the TiO_2_ conduction band. Charging of TiO_2_ became evident from the large photocathodic charging spikes and anodic current response in the dark phase of the light‐chopped LSV scans. The ratio of the photocathodic to anodic charge is indeed close to unity for Si|IO‐TiO_2_, indicating negligible catalytic turnover. In contrast, the cathodic charge is far higher than the anodic response for H_2_ase‐modified electrodes, which supports efficient interfacial charge transfer and catalytic turnover at the enzyme.

**Figure 2:**
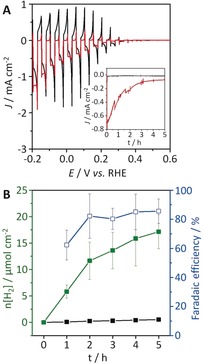
A) LSV scans of Si|IO‐TiO_2_ (black) and Si|IO‐TiO_2_|H_2_ase (red) at a scan rate of 5 mV s^−1^ under chopped‐light irradiation (100 mW cm^−2^; AM1.5G; IR water filter; *λ*>420 nm; 25 °C). Inset: CPPE of the electrodes at 0.0 V vs. RHE. B) Time profiles of H_2_ production (green) and the corresponding Faradaic efficiency (blue) during 5 h of CPPE at 0.0 V vs. RHE under visible‐light irradiation for Si|IO‐TiO_2_|H_2_ase. The H_2_ production for Si|IO‐TiO_2_ (black) is also shown for comparison (see also Figure S9 C). Conditions: 50 mm of MES solution (pH 6.0) containing 50 mm KCl, N_2_ atmosphere, room temperature, geometrical surface area: 0.178 cm^2^ for all electrodes.

Controlled potential photoelectrolysis (CPPE) with Si|IO‐TiO_2_ at 0.0 V vs. RHE showed a photocurrent close to zero after less than one minute (Figure 2 A, inset; Figure S9 B), and only a small amount of H_2_ (0.5±0.1 μmol cm^−2^) with a modest Faradaic efficiency (45±9 %) was produced during 5 h (Figures 2 B and S9 C). In contrast, Si|IO‐TiO_2_|H_2_ase maintained good photocathodic currents during 5 h of CPPE (Figure 2 A, inset), and headspace gas analysis by gas chromatography revealed the generation of 17±3 μmol cm^−2^ of H_2_ with a Faradaic efficiency of (86±8) %. The H_2_ase is therefore electroactive and relatively robust in the IO‐TiO_2_ scaffold. Control experiments in the presence of Pt nanoparticles instead of H_2_ase showed comparable electrochemical responses (Figures S10 and S11).

The Si|IO‐TiO_2_|H_2_ase photocathode was then paired with photoanodes. Previously, the H_2_ evolving *Clostridium acetobutylicum* [FeFe] hydrogenase HydA, adsorbed on a pyrolytic graphite edge electrode, had been connected to a porphyrin‐sensitized TiO_2_ photoanode. This PEC cell relied on the consumption of sacrificial NADH (nicotinamide adenine dinucleotide),1d whereas we demonstrate overall water splitting in this work. BiVO_4_ is a well‐established photoanode for water oxidation,^9^ which was synthesized on FTO‐coated glass according to previous reports.^10^ BiVO_4_ was selected owing to its stability under the neutral pH conditions required for the H_2_ase, and its high photovoltage and currents are in principle suitable for bias‐free water splitting when paired with a silicon photocathode.^9^, 10a,10b The synthesized BiVO_4_ is crystalline and exhibits a film thickness of approximately 650 nm with a nanoporous surface structure (see Figure S12 for SEM images, XRD pattern, and UV/Vis spectrum). The kinetics of water oxidation was enhanced by deposition of a molecular TiCo precatalyst on the BiVO_4_ surface from a single source precursor as previously reported.10b, 11

FTO|BiVO_4_|TiCo exhibited a photocurrent of 1.0 mA cm^−2^ at 1.23 V vs. RHE in MES/KCl solution (50 mm each, pH 6.0) for UV‐filtered simulated solar light irradiation (100 mW cm^−2^; AM1.5G; IR water filter; *λ*>420 nm; 25 °C), driving water oxidation with high stability during the 5 h of CPPE (Figure S13). Comparison of the LSV scans of FTO|BiVO_4_|TiCo and Si|IO‐TiO_2_|H_2_ase obtained from three‐electrode measurements showed a photocurrent of approximately 15 μA at the intersection of both voltammetric scans (0.18 V vs. RHE), suggesting the feasibility of unassisted water splitting in a tandem PEC cell (Figure 3 A).

**Figure 3:**
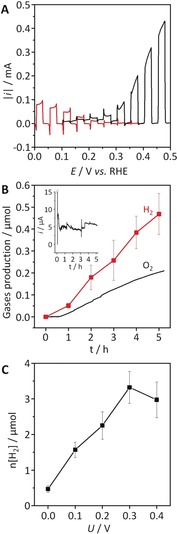
A) LSV scans of FTO|BiVO_4_|TiCo (black) and Si|IO‐TiO_2_|H_2_ase (red) obtained from three‐electrode measurements (note that the current for Si|IO‐TiO_2_|H_2_ase was inverted for ease of comparison). B) Time profiles of H_2_ and O_2_ production during unassisted solar water splitting in a two‐electrode PEC cell with Si|IO‐TiO_2_|H_2_ase wired to FTO|BiVO_4_|TiCo in a two‐electrode configuration. The inset shows the *I*–*t* trace from the CPPE measurement. C) Total amount of H_2_ produced during 5 h PEC water splitting as a function of the applied voltage. In all experiments, the geometrical surface areas of FTO|BiVO_4_|TiCo and Si|IO‐TiO_2_|H_2_ase were 4 and 0.178 cm^2^, respectively. Conditions: Visible‐light irradiation (100 mW cm^−2^; AM1.5G; IR water filter; *λ*>420 nm; 25 °C), 50 mm of MES solution (pH 6.0) containing 50 mm KCl, N_2_ atmosphere, room temperature.

Thus a two‐electrode configuration was adapted with a Nafion membrane separating the anodic from the cathodic compartment. Irradiation of the two‐electrode tandem PEC cell without an external voltage (*U*=0 V) for five hours gave a constant photocurrent profile and generated 0.47±0.09 μmol of H_2_ and 0.20 μmol of O_2_, which corresponds to Faradaic efficiencies of 98±14 % and 84 %, respectively (Figure 3 B). The performance of the tandem PEC cell was also studied with different external voltages (Figures 3 C and S14). As expected, the photocurrent and the quantity of H_2_ increased with higher voltages, maintaining a Faradaic efficiency of more than 80 % after 5 h CPPE in all measurements.

The Si|IO‐TiO_2_|H_2_ase photocathode was subsequently paired with the biological water oxidation photocatalyst PSII (isolated from *Thermosynechococcus elongatus*) immobilized on an anode. H_2_ase had previously been wired to PSII in a PEC configuration, but both enzymes were immobilized on hierarchical IO‐ITO electrodes.3a The single light‐absorbing PEC cell contained a “dark” IO‐ITO|H_2_ase cathode, which resulted in the requirement of a large external voltage (*U*>0.6 V) to achieve overall water splitting. Our Si|IO‐TiO_2_|H_2_ase electrode provides a unique opportunity to reduce this thermodynamic barrier needed for overall water splitting using H_2_ase and PSII.

The energetics of the electrons afforded by the IO‐ITO|PSII anode is dependent on the terminal electron acceptors within PSII (quinones Q_A_ and Q_B_). To minimize energy loss of the electrons leaving PSII, a number of soluble Q_B_ mimics with more negative redox potentials than the commonly employed mediator 2,6‐dichloro‐1,4‐benzoquinone (DCBQ, *E*_m_=329 mV vs. NHE)^12^ were studied. Although 2,6‐di‐*tert*‐butyl‐1,4‐benzoquinone (DTBpQ, *E*_m_=92 mV vs. NHE) showed the most negative onset potential, it also exhibited low aqueous solubility, giving rise to lower overall photocurrents. As such, 3,5‐di‐*tert*‐butyl‐1,2‐benzoquinone (DTBoQ, *E*_m_=290 mV vs. NHE) was identified as the most suitable redox shuttle to mediate charge at the PSII–ITO interface as it gives rise to a 100 mV earlier photocurrent onset than DCBQ (Figure S15). Comparing a stepped chronoamperometry scan of FTO|IO‐ITO|PSII (90 pmol PSII; Figure S16) in the presence of DTBoQ with an LSV scan of Si|IO‐TiO_2_|H_2_ase shows that an applied voltage of *U*>0.24 V will be required for solar PEC water splitting (Figures 4 A and S17). A two‐electrode PEC cell consisting of the FTO|IO‐ITO|PSII photoanode coupled to the Si|IO‐TiO_2_|H_2_ase photocathode was irradiated with UV‐filtered simulated solar light (100 mW cm^−2^; AM1.5G; IR water filter; *λ*>420 nm; 25 °C) for 3 h at *U*=0.4 V, which resulted in the generation of 0.70±0.13 μmol cm^−2^ of H_2_ with a Faradaic efficiency of (91±19) % (Figure 4 B). This semi‐artificial tandem PEC cell therefore allows for a wider usage of the solar spectrum at a reduced voltage than the previously reported3a single‐light‐absorber system.

**Figure 4:**
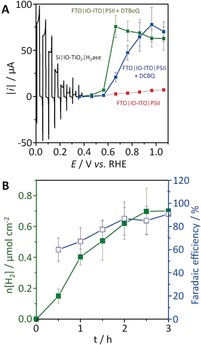
A) Stepped chronoamperometry scans of FTO|IO‐ITO|PSII without a soluble redox mediator (red), with DCBQ (blue), and with DTBoQ (green). An LSV scan of Si|IO‐TiO_2_|H_2_ase (black) with inverted current is also shown. The loading amounts of PSII and H_2_ase were 90 and 80 pmol, respectively. All scans were carried out in a three‐electrode configuration under chopped light irradiation. B) Time profiles of H_2_ production (green) and the corresponding Faradaic efficiency (blue) during two‐electrode PEC water splitting of FTO|IO‐ITO|PSII with DTBoQ wired to Si|IO‐TiO_2_|H_2_ase at an applied voltage of 0.4 V. In all experiments, the geometrical surface areas of FTO|IO‐ITO|PSII and Si|IO‐TiO_2_|H_2_ase were 0.5 and 0.178 cm^2^, respectively. Conditions: Simulated solar light (100 mW cm^−2^; AM1.5G; IR water filter; *λ*>420 nm; 25 °C), 50 mm of MES solution (pH 6.0) containing 50 mm KCl, 1 mm of Q_B_ mimics, N_2_ atmosphere, room temperature.

In summary, we have developed a hierarchically structured photocathode and demonstrated by PEC, QCM, and ATR‐IR analysis its excellent and stable integration of an electroactive H_2_ase. This Si|IO‐TiO_2_|H_2_ase photocathode is a platform for the production of H_2_ from tandem PEC water spitting with photoanodes. Using a BiVO_4_ photoanode enabled stable and unassisted solar water splitting with a hydrogenase in vitro. Pairing of the H_2_ase photocathode with a PSII photoanode allows tandem water splitting with wired enzymes in an engineered Z‐scheme for complementary light absorption. The presented semi‐artificial platform is suitable for the integration of a wide range of biological catalysts and guests in the future.

## Conflict of interest

The authors declare no conflict of interest.

## Supporting information

As a service to our authors and readers, this journal provides supporting information supplied by the authors. Such materials are peer reviewed and may be re‐organized for online delivery, but are not copy‐edited or typeset. Technical support issues arising from supporting information (other than missing files) should be addressed to the authors.

Supplementary
