# Supplementary material for: Solar Water Splitting with a Hydrogenase Integrated in Photoelectrochemical Tandem Cells
Source: Angew Chem Int Ed Engl. 2018 Jul 17;57(33):10595–9. doi: 10.1002/anie.201805027 (PMC6100105; doi:10.1002/anie.201805027)
Supplement: Supplementary file 1 — Supplementary [file ANIE-57-10595-s001.pdf]

Angewandte Chemie
Eine Zeitschrift der Gesellschaft Deutscher Chemiker

Supporting Information

Solar Water Splitting with a Hydrogenase Integrated in Photoelectrochemical Tandem Cells

*Dong Heon Nam, Jenny Z. Zhang, Virgil Andrei, Nikolay Kornienko, Nina Heidary, Andreas Wagner, Kenichi Nakanishi, Katarzyna P. Sokol, Barnaby Slater, Ingo Zebger, Stephan Hofmann, Juan C. Fontecilla-Camps, Chan Beum Park, and Erwin Reisner**

anie_201805027_sm_miscellaneous_information.pdf

Experimental Section

Materials. All chemicals were purchased from commercial suppliers and used without further purification unless otherwise noted. Reagents for the analytical part of the work were of the highest available purity. [NiFeSe]-hydrogenase from *Desulfomicrobium baculatum* was purified and characterized as reported previously.[1] PSII was isolated from the thermophilic cyanobacterium *Thermosynechococcus elongatus* and purified according to a previously reported procedure, resulting in purified PSII with an average oxygen-evolving activity of approximately 5300 μmol O_2 h^{-1} mg^{-1} of chlorophyll *a* (Chl *a*).[2]

***p*-Si protected with ALD-coated TiO_2.** The *p*-type boron-doped silicon wafers (resistivity of 1-10 ohm cm; <100>; 500 μm thickness; single-side polished, University Wafer) were cleaned in isopropanol, blown dry with N_2, etched for 2 min in buffered oxide etch (6:1 volume ratio of 40% NH_4F in water and 49% HF), rinsing in water and drying under N_2. The samples were loaded at 60 °C into the ALD chamber (Cambridge Nanotech Savannah S100 G1), which was immediately evacuated to prevent oxide formation, before being ramped to 200 °C. TiO_2 was deposited in a multi-pulse mode at 200 °C as described in detail elsewhere.[3] Titanium(IV) isopropoxide (TTIP), volatilized at 90 °C, was used as the precursor and deionized H_2O vapor as the oxidant. Both TTIP and H_2O doses were set equally at 1.45-1.65 Torr.s. The precursor and oxidant were delivered sequentially to the reaction chamber separated by 15 s of purging with 20 sccm of N_2 to prevent a CVD-like reaction. TTIP was pulsed first in the sequence to minimize any initial oxidation. The total number of cycles was set to 115 to achieve the ALD of approximately 4 nm thick TiO_2 layers.

Si|IO-TiO_2|H_2ase photocathode. The inverse opal TiO_2 (IO-TiO_2) layer was assembled on top of the Si|ALD-TiO_2 surface (1 cm x 2 cm) following a previously reported co-assembly procedure for hierarchical indium-tin oxide.[4] Briefly, TiO_2 nanoparticles (P25, 36 mg) were dispersed by sonication in a MeOH/water (4:1 v/v) mixture (360 μL) for 3 h. The dispersion of polystyrene beads (1.2 mL; 2.54% w/v suspension in water; 750 nm bead diameter) was centrifuged to remove the supernatant. The P25 nanoparticle solution was added to the polystyrene pellet, and sonicated for 5 min in ice-cold water (< 5 °C) to give the polystyrene-P25 dispersion. The polystyrene-P25 dispersion (5 μL) was drop-cast onto the Si|TiO_2 surface (0.178 cm^2 geometrical surface area) to fabricate a 10 μm thickness of IO-TiO_2 layer. The electrodes were sintered at 450 °C for 2 h (ramping rate: 1 °C min^{-1}). To immobilize H_2ase on the Si|TiO_2|IO-TiO_2 electrode, 8 μM of H_2ase solution (10 μL) was drop-cast onto a UV-ozone treated electrode. The electric contact was formed using a Ga:In eutectic solution

(99.99%, Sigma-Aldrich) and a copper wire covered with silver paste and epoxy resin. For Pt deposition instead of H_2ase, Si|TiO_2|IO-TiO_2 was immersed in phosphate buffer (100 mM; pH 6.0; 7 mM of K_2PtCl_4; 7 mM of ascorbic acid), and irradiated with visible light (λ > 400 nm) for 1 h, followed by washing with excess water.

FTO|BiVO$_4$|TiCo photoanode. FTO|$BiVO_4$ (4 cm^2 geometrical surface area) was synthesized following a published procedure,[5] and the TiCo pre-catalyst was deposited via single source precursor chemistry as previously reported.[5b,6] Briefly, 50 mL of a $Bi(NO_3)_3 \cdot 5H_2O$ (0.02 M) and NaI (0.4 M) aqueous solution was sonicated and acidified to pH 1.20, before it was added to a sonicated p-benzoquinone (0.3 M) solution in ethanol (22.5 mL). BiOI was electrodeposited from the resulting mixture onto the Piranha cleaned FTO-coated glass substrate, by applying –0.3 V against a Ag/AgCl reference electrode for 5 s, then –0.1 V for 180 s. 40 µL cm^{-2} of a 0.4 M $VO(acac)_2$ solution in DMSO were drop-cast onto the electrodes before annealing at 450 °C for 1 h (ramping rate: 1 °C min^{-1}). The excess V_2O_5 was dissolved by stirring in a 0.2 M NaOH solution. The TiCo pre-catalyst was deposited by spin coating 20 µL cm^{-2} of a cobalt-containing polyoxotitanate ([$Ti_4O(OEt)_{15}(CoCl)$]) solution (4.8 mg mL^{-1} in dry toluene) four times in air (2000 RPM, 10 s).

FTO|IO-ITO|PSII photoanode. FTO|IO-ITO electrodes (macropore diameter of 750 nm; 0.5 cm^2 geometrical surface area; 10 µm thickness of IO-ITO layer) were fabricated according to a previously reported co-assembly procedure.[4] To immobilize PSII onto the FTO|IO-ITO electrode, a PSII stock solution (90 pmol) was drop-cast onto an UV-ozone treated electrode, and incubated in the dark for 15 min at room temperature. The amount of PSII on the IO-ITO surface was quantified by UV-vis spectrophotometry of Chl *a*, which was carried out by scratching off the IO-ITO from the FTO glass followed by washing with MeOH to extract Chl *a* from the IO-ITO. The band with an absorption maximum of λ_{max} = 665 nm assigned to Chl *a* (extinction coefficient ε = 79.95 (Chl *a* mg)$^{-1}$ mL cm^{-1}) was used to calculate the amount of PSII monomers assuming 35 Chl *a* molecules per PSII monomer.[7]

Quartz crystal microbalance (QCM) analysis. QCM experiments were conducted in a 50 mM MES buffer solution (pH 6) containing 50 mM KCl using a Biolin Scientific Q-Sense Explorer instrument. The piezoelectric AT-cut quartz chips were utilized for quantification of H_2ase loading on different TiO_2 architectures. To analyze a planar TiO_2, the quartz chip covered with a planar TiO_2 surface was used as a mass sensor without any modification. For mesoporous TiO_2, 20 wt% of P25 nanoparticles in ethanol with 5 M acetic acid was drop-cast

onto the quartz chip with a planar gold surface, which was followed by sintering at 450 °C for 20 min with 1 °C min^{-1} ramping rate. The inverse opal TiO_2 was deposited onto the quartz chip with a planar gold surface following a previously reported co-assembly procedure.[4] The thickness and morphology of these TiO_2 architectures were determined by scanning electron microscopy (TESCAN MIRA3 FEG-SEM). Throughout the measurement, the entire system was purged with N_2 gas. A total volume of 5 mL was used and following signal stabilization, H_2ase was injected to achieve a total concentration of 5 nM. The mass loading was estimated from the frequency values using the Sauerbrey Equation: $\Delta f = -\frac{2f_0^2}{A\sqrt{p_q \mu_q}}\Delta m$, where f_0 is the resonance frequency of the quartz oscillator, A is the piezoelectrically active crystal area, Δm is the change in mass, p_q is the density of quartz, and μ_q is the shear modulus of quartz. The loading quantity of hydrogenase was estimated by converting the Sauerbrey mass to mols of enzyme (87.4 kDa) with the additional assumption that 25% of the adsorbed mass is attributed to water bound to the enzyme.

ATR-IR analysis. Spectroelectrochemical ATR-IR measurements were performed in a single-reflection PIKE ATR-IR setup and a customized ATR-cell using a Si prism with an angle of incidence of 60°. A 10 μm thickness of IO-TiO_2 film was deposited on the flat Si surface to probe H_2ase immobilization. ATR-IR spectra were recorded from 4000 to 1000 cm^{-1} with a spectral resolution of 4 cm^{-1} on a Bruker Vertex 70 spectrometer equipped with a photovoltaic MCT detector. Two hundred scans were co-added for one spectrum, requiring an accumulation time of 1.5 min. ATR-IR spectra were evaluated using the OPUS 5.5 software. Immobilization of the H_2ase was accomplished by incubating a 8 μM of H_2ase solution (10 μL) added to a 1 mL of Tris buffer solution (20 mM) at pH 7.6 (pre-equilibrated and N_2-purged inside the ATR-cell) for 30 min at room temperature.

Characterization. The morphology of the electrodes was studied by scanning electron microscopy (TESCAN MIRA3 FEG-SEM) and atomic force microscopy (Oxford Instruments MFP-3D Classic). X-ray diffraction (XRD) analysis was carried out using a EMPYREAN Series 2 X-ray diffractometer, and UV/Vis absorption spectra were measured by a Varian Cary 50 Bio UV-vis spectrometer. An Agilent 7890A series gas chromatograph equipped with a 5 Å molecular sieve column and N_2 carrier gas was employed for the quantification of H_2. Quantification of O_2 was performed with a calibrated fluorescence-based O_2 sensor (Neofox with an Ocean Optics FOSPHOR probe).

Electrochemical studies. All electrochemical experiments were performed with an Ivium CompactStat potentiostat. The electrolyte solution for all experiments contained 50 mM MES buffer (2-(N-morpholino)ethanesulfonic acid; pH 6.0) and 50 mM KCl. For the mediated photocurrent measurements of FTO|IO-ITO|PSII, a 3,5-ditertbutyl-1,2-benzoquinone (DTBoQ) solution in DMSO (100 mM) was added to give a final concentration of 1 mM in the electrolyte solution. A Newport Oriel Xenon 150 W solar light simulator (100 mW cm^{-2}; AM1.5G; IR water filter; $\lambda > 420$ nm UV cut-off filter) was used as the light source. In the three-electrode configuration, an Ag/AgCl (3M KCl) electrode and a platinum mesh were used as reference and counter electrodes, respectively. All redox potentials are quoted against the reversible hydrogen electrode (RHE), and the potentials were obtained by using the following correction factor: $E_{\mathrm{RHE}} = E_{\mathrm{Ag/AgCl}} + 0.209\ \mathrm{V} + 0.059\times\mathrm{pH}$. To perform solar water splitting, a two-electrode configuration was employed in a gas-tight two-compartment cell with a Nafion membrane separating the compartments.

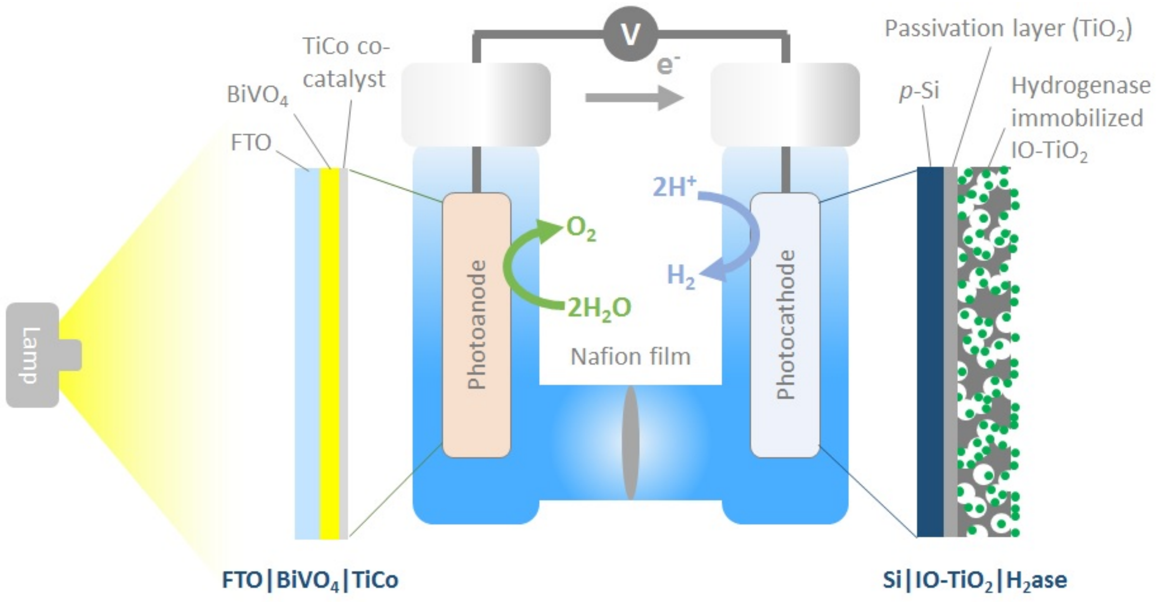

Figure S1. Schematic illustration of unassisted solar water splitting performed by a photoelectrochemical (PEC) cell consisting of a FTO|$BiVO_4$|TiCo photoanode and a Si|IO-TiO_2|H_2ase photocathode. Under visible light illumination, $BiVO_4$ absorbs light and generates a driving force for water oxidation catalyzed by a TiCo catalyst. Photoexcitation of p-Si generates low potential electrons for proton reduction at the hydrogenase (H_2ase).

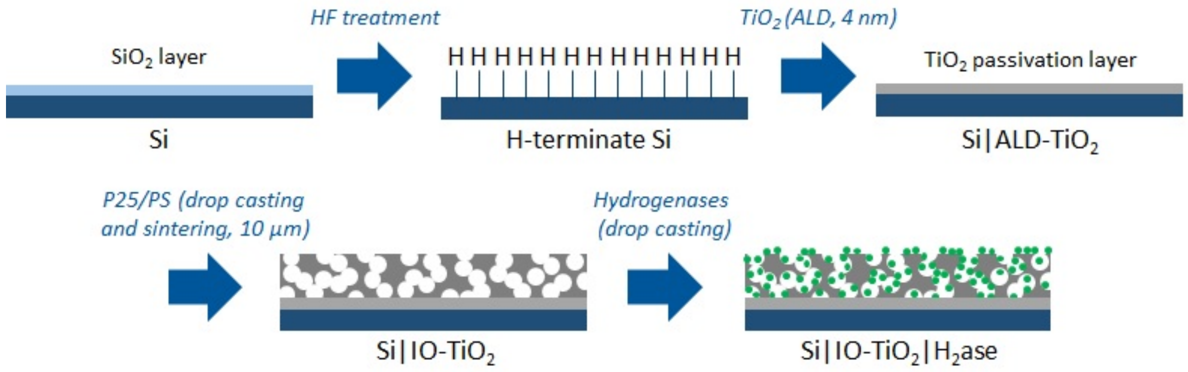

Figure S2. Fabrication of bespoke Si|IO-TiO_2|H_2ase photocathode. The *p*-Si wafer was sequentially cleaned with isopropanol and hydrofluoric acid (HF) to remove inorganic/organic impurities and native oxide layer on Si wafer surface. To improve the long-term stability of Si, thin TiO_2 layer (4 nm) was deposited on Si wafer surface immediately after HF treatment by atomic layer deposition (ALD). The inverse opal TiO_2 (IO-TiO_2) layer was prepared on Si|ALD-TiO_2 surface by a co-assembly procedure with TiO_2 nanoparticles and polystyrene beads. To immobilize hydrogenase (H_2ase) on Si|IO-TiO_2, one aliquot of H_2ase solution (80 pmol) was drop-cast onto UV-ozone treated electrode.

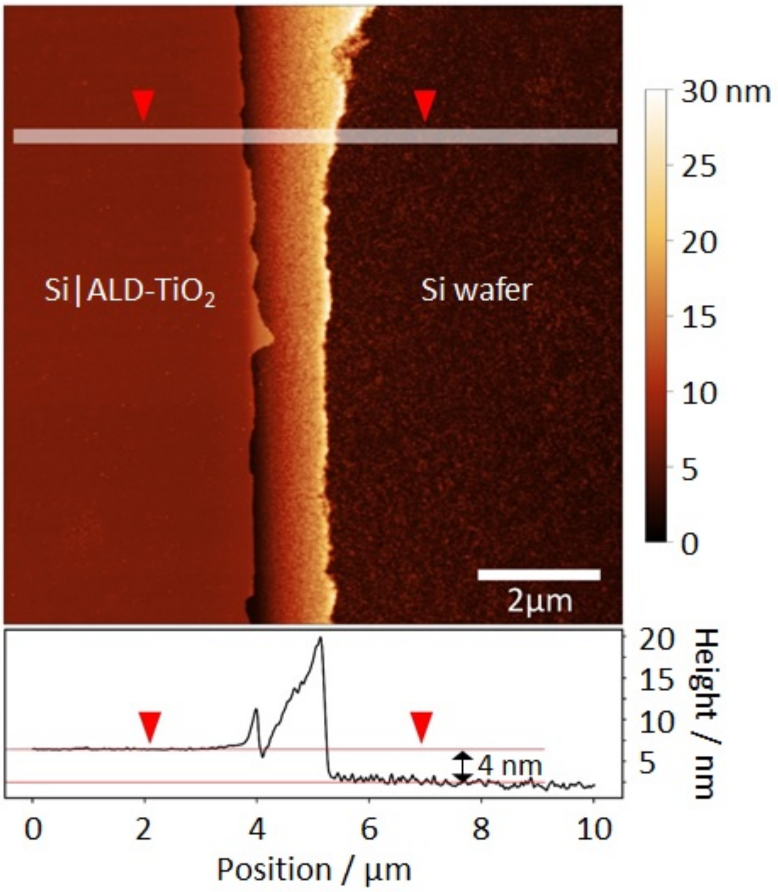

Figure S3. Atomic force microscopy (AFM) image of Si|ALD-TiO_2 showing a thin TiO_2 layer (4 nm) deposited on the *p*-Si wafer surface. Note that the boundary between Si|ALD-TiO_2 and Si wafer exhibited a high height around 20 nm due to the marker ink.

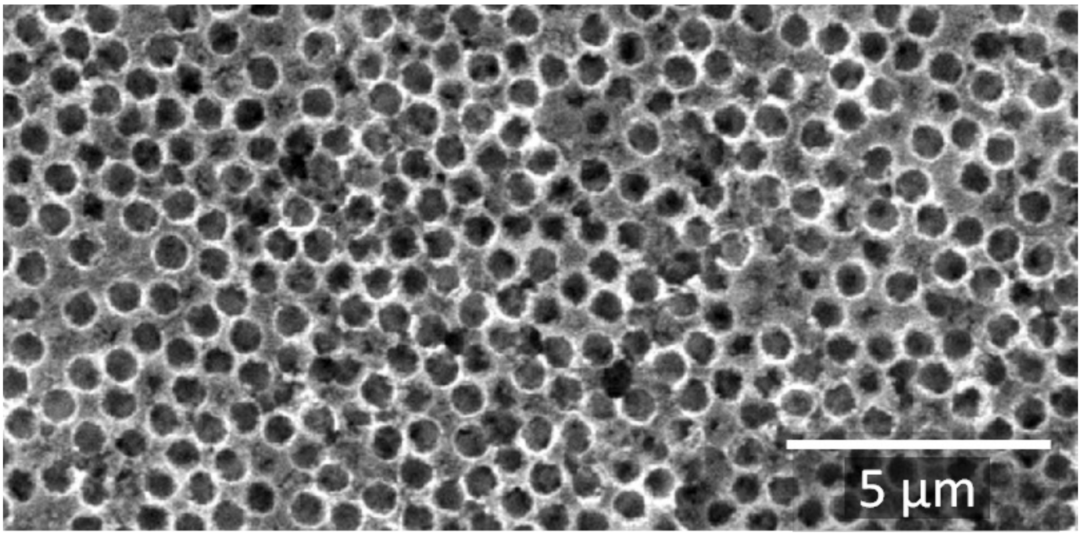

Figure S4. Top scanning electron microscopy (SEM) images of the Si|IO-TiO_2 photocathode.

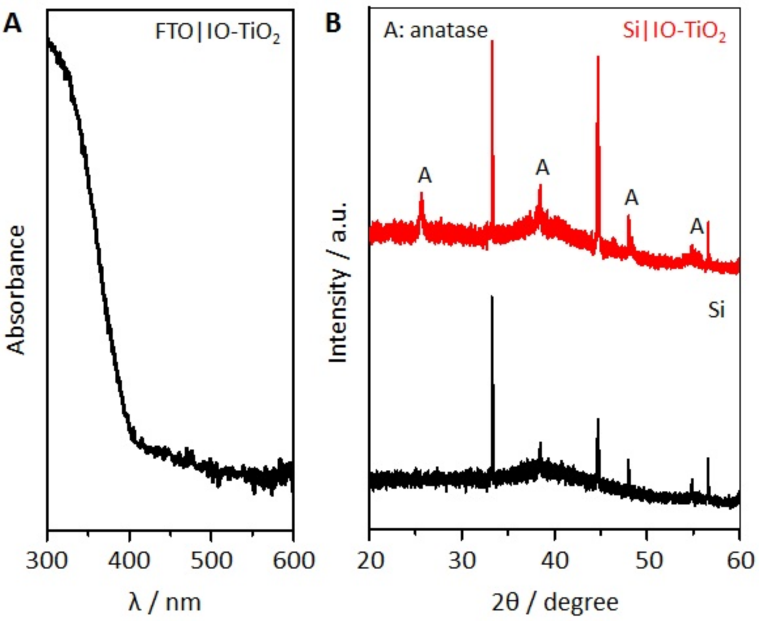

Figure S5. (A) The UV-vis spectrum of FTO|IO-TiO_2. (B) X-ray diffraction (XRD) patterns of Si (black trace) and Si|IO-TiO_2 (red trace).

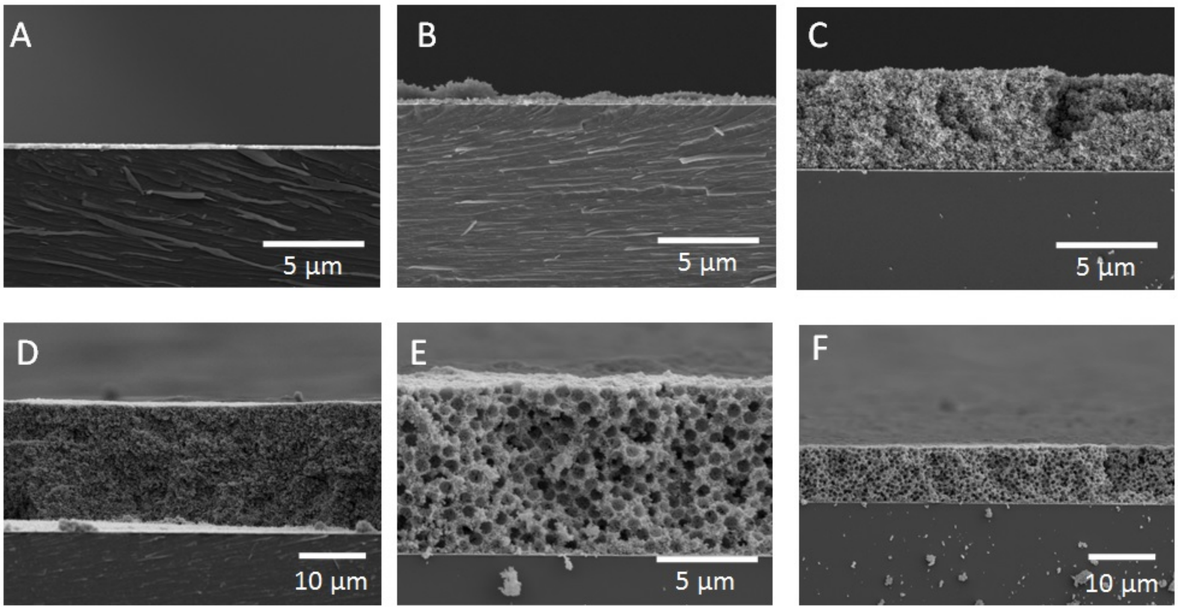

Figure S6. Cross-sectional SEM imaging of the electrode architectures used for QCM studies. H_2ase adsorption onto (A) planar TiO_2 was compared to that on (B) 0.4 µm mesoporous, (C) 4 µm mesoporous, (D) 17 µm mesoporous, and (E, F) 7 µm IO-TiO_2.

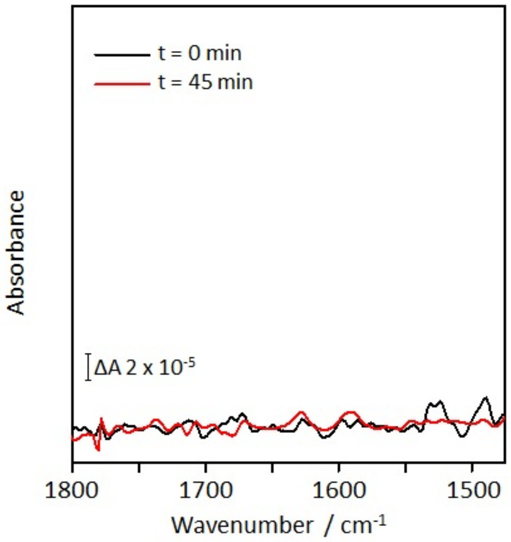

Figure S7. ATR-IR spectra of Si prism|meso-TiO_2|H_2ase during H_2ase incubation with 80 pmol H_2ase after 0 and 45 min. Although immobilization of the H_2ase was accomplished by incubating a 8 µM of H_2ase (10 µL) solution when added to a 1 mL of Tris buffer solution (20 mM) at pH 7.6 (pre-equilibrated and N_2-purged inside the ATR-cell) for 45 min at room temperature, no spectral signature of the enzyme was detectable. This is related to the restricted penetration depth of the evanescent wave into the bottom of the 1 µm thick meso-TiO_2, which is only approximately 0.5 µm from the ATR-Si prism surface.

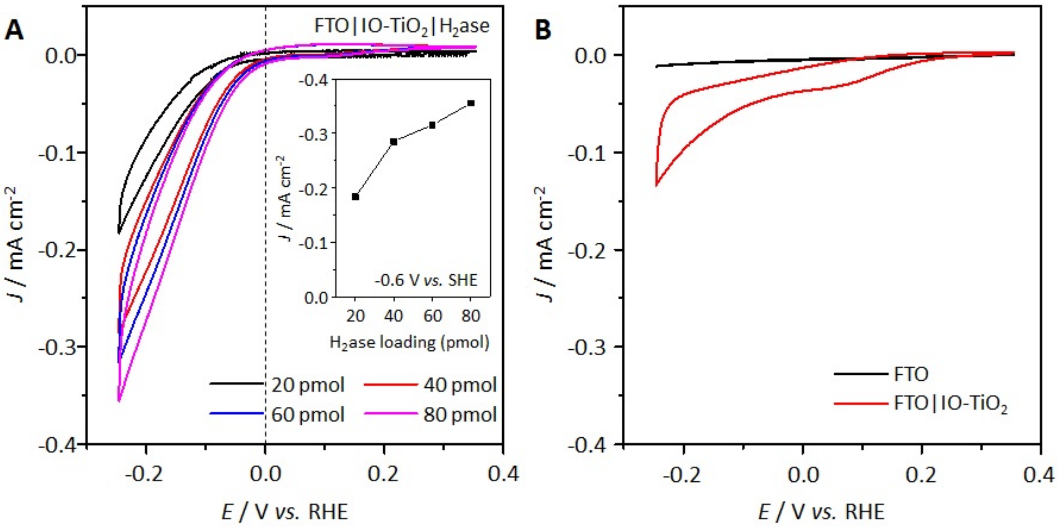

Figure S8. (A) Protein film voltammetry scans of FTO|IO-TiO_2|H_2ase showing the electrocatalytic response for proton reduction with different H_2ase loading. The inset shows the catalytic current at −0.6 V *vs.* SHE (*i.e.*, −0.246 V *vs.* RHE) as a function of H_2ase loading. (B) Cyclic voltammetry scans of FTO (black trace) and FTO|IO-TiO_2 (red trace) as control experiments. Conditions: 50 mM of MES buffer (pH 6.0) containing 50 mM KCl, N_2 atmosphere, room temperature, 5 mV s^{-1} scan rate, 0.178 cm^2 of geometrical surface area for all electrodes.

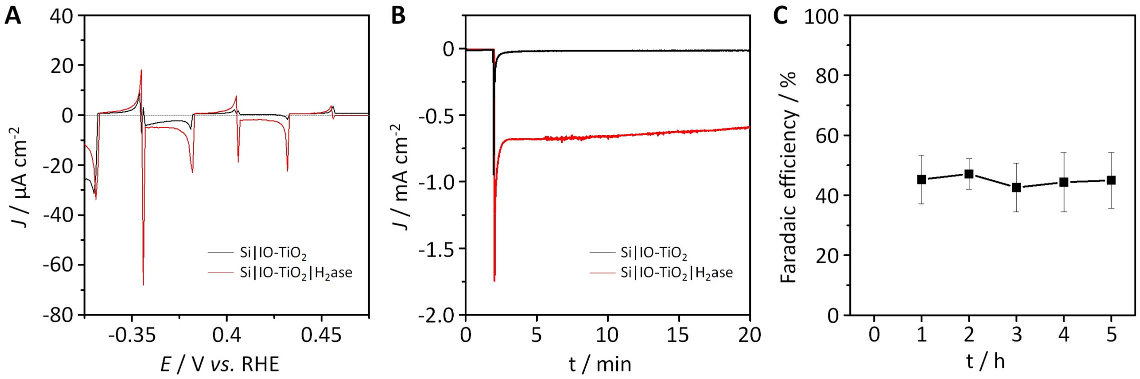

Figure S9. (A) LSV scans (scan rate: 5 mV s^{-1}) and (B) controlled potential photoelectrolysis (at 0.0 V *vs.* RHE) of Si|IO-TiO_2|H_2ase (red trace) and Si|IO-TiO_2 (black trace). (C) Faradaic efficiency for H_2 production of Si|IO-TiO_2 during 5 h of controlled potential photoelectrolysis at 0.0 V *vs.* RHE. All electrochemical analyses performed under visible light irradiation (100 mW cm^{-2}; AM1.5G; IR water filter; λ > 420 nm; 25 ºC). The electrolyte solution (pH 6.0) contained 50 mM MES and 50 mM KCl, and the geometrical surface area of the electrode was 0.178 cm^2.

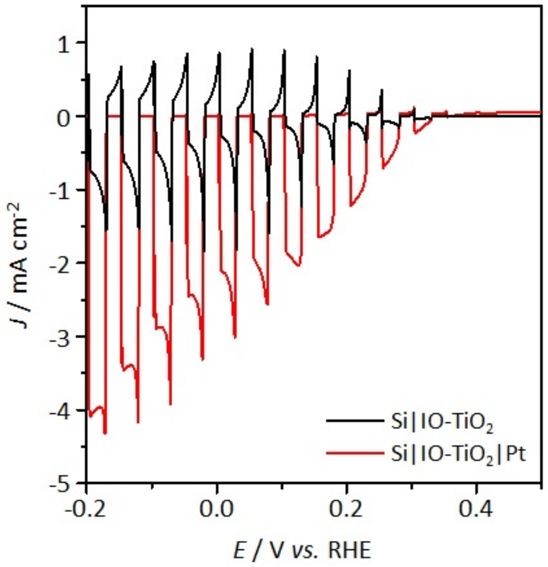

Figure S10. Linear sweep voltammetry scans of Si|IO-TiO_2 (black trace) and Si|IO-TiO_2|Pt (red trace) with 5 mV s^{-1} scan rate under chopped light (100 mW cm^{-2}; AM1.5G; IR water filter; λ > 420 nm UV filter). Conditions: 50 mM of MES buffer (pH 6.0) containing 50 mM KCl, under N_2 atmosphere, room temperature, 0.178 cm^2 of geometrical surface area for all electrodes.

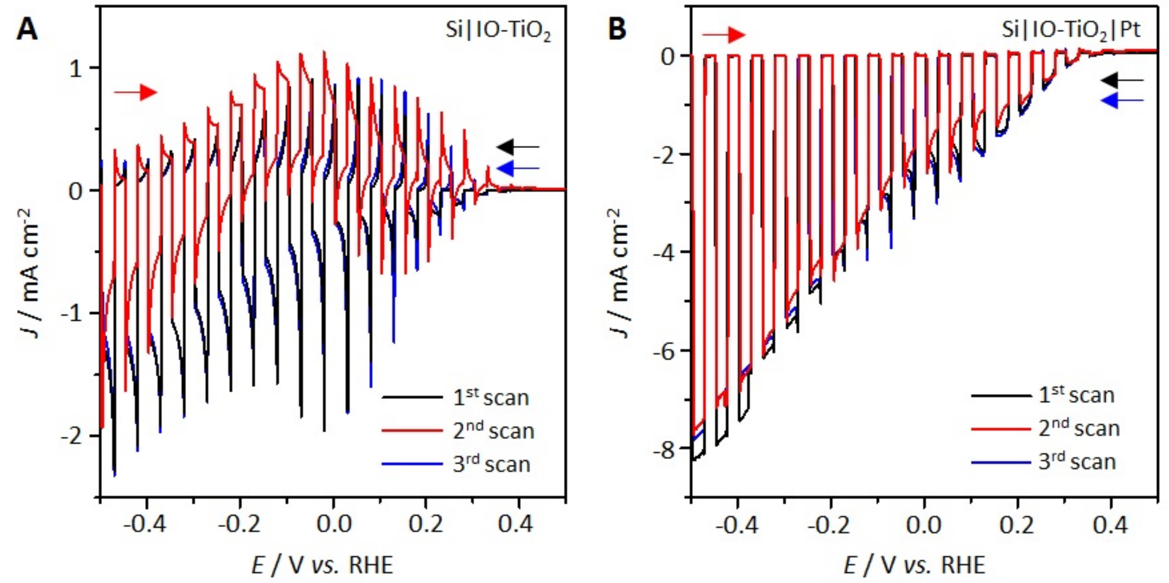

Figure S11. Consecutive linear sweep voltammetry (LSV) scans of (A) Si|IO-TiO_2 and (B) Si|IO-TiO_2|Pt with a 5 mV s^{-1} scan rate under chopped light (100 mW cm^{-2}; AM1.5G; IR water filter; λ > 420 nm UV filter). No anodic discharging features (i.e., no charge accumulation) were observed after Pt deposition on Si|IO-TiO_2. Conditions: 50 mM of MES buffer (pH 6.0) containing 50 mM KCl, N_2 atmosphere, room temperature, 0.178 cm^2 of geometrical surface area for all electrodes.

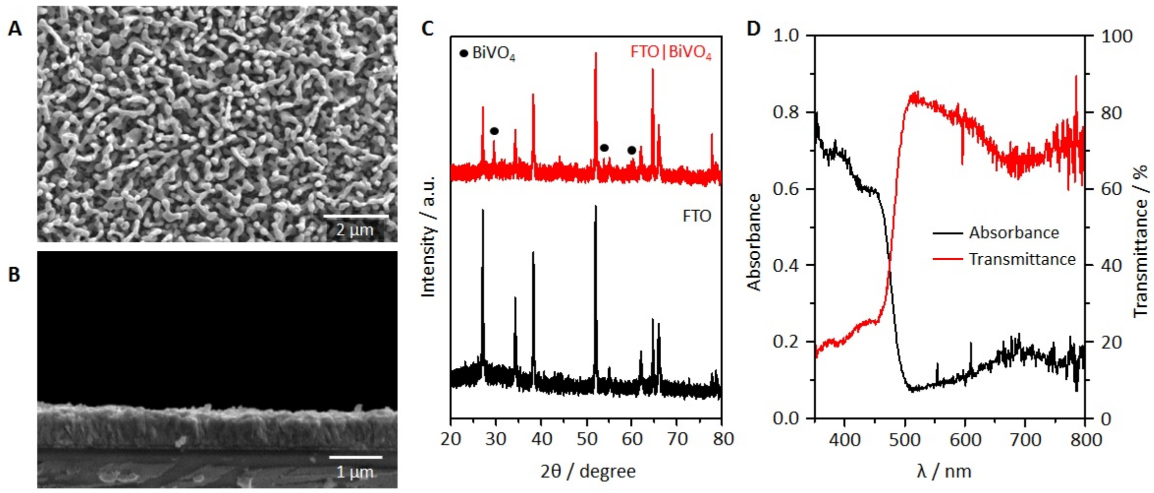

Figure S12. (A) Top and (B) cross-sectional SEM images of the FTO|$BiVO_4$ photoanode showing an approximately 650 nm-thick nanoporous surface structure. (C) X-ray diffraction (XRD) patterns of FTO (black trace) and FTO|$BiVO_4$ (red trace) matched well with the reference (JCPDS #14-0688). (D) UV-vis and transmittance spectra of FTO|$BiVO_4$.

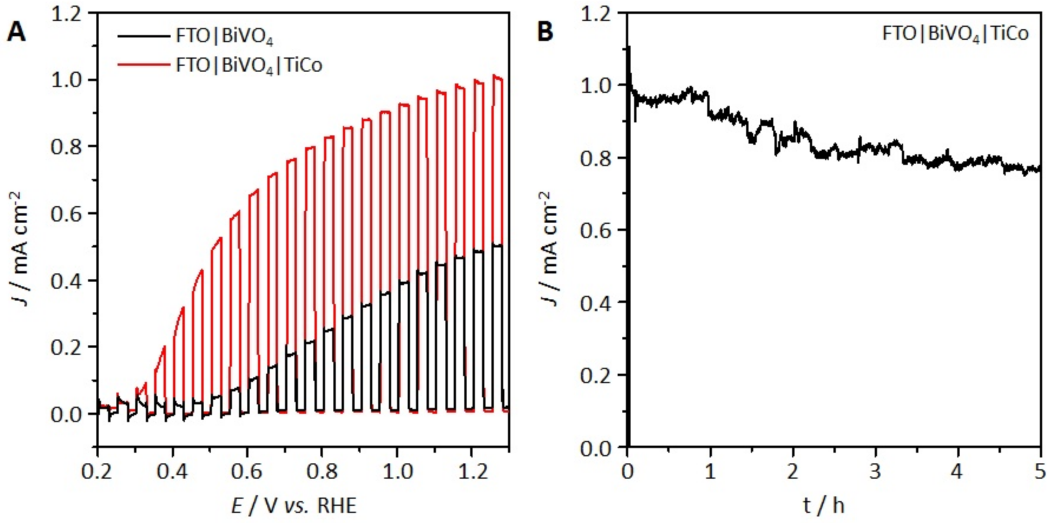

Figure S13. (A) Linear sweep voltammetry (LSV) scans of FTO|$BiVO_4$ (black trace) and FTO|$BiVO_4$|TiCo (red trace) with 5 mV s^{-1} scan rate under chopped light (100 mW cm^{-2}; AM1.5G; IR water filter; λ > 420 nm UV filter). (B) Controlled potential photoelectrolysis (CPPE) of FTO|$BiVO_4$|TiCo at 1.23 V *vs.* RHE under visible light irradiation (100 mW cm^{-2}; AM1.5G; IR water filter; λ > 420 nm UV filter), exhibiting good photocurrent and stability of FTO|$BiVO_4$|TiCo for water oxidation. Conditions: 50 mM of MES buffer (pH 6.0) containing 50 mM KCl, N_2 atmosphere, room temperature, 4 cm^2 of geometrical surface area for all electrodes.

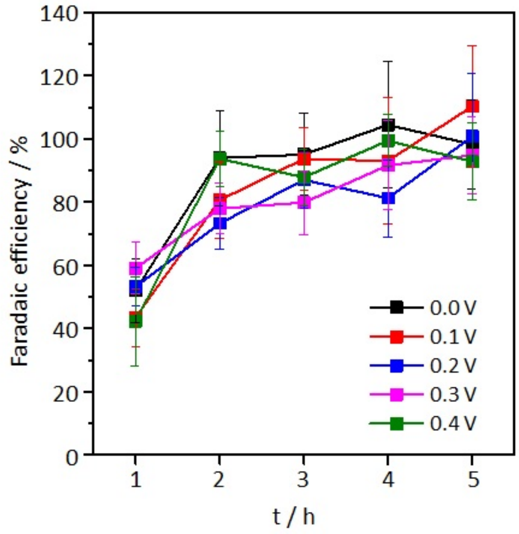

Figure S14. Faradaic efficiencies of H_2 production with FTO|$BiVO_4$|TiCo and Si|IO-TiO_2|H_2ase (two-electrode configuration) at various applied bias voltages. The low values of Faradaic efficiency at the first hour can be attributed to slow H_2 diffusion to the headspace by H_2 trapping in the pores of the IO-TiO_2 scaffold. The geometrical surface areas of FTO|$BiVO_4$|TiCo and Si|IO-TiO_2|H_2ase are 4 and 0.178 cm^2, respectively. Conditions: visible light irradiation (100 mW cm^{-2}; AM1.5G; IR water filter; λ > 420 nm UV filter), 50 mM of MES buffer (pH 6.0) containing 50 mM KCl, N_2 atmosphere, room temperature.

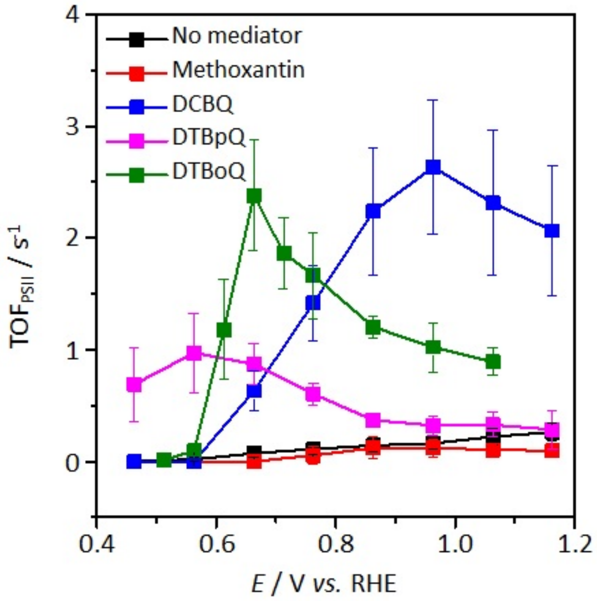

Figure S15. Stepped chronoamperometry scans of FTO|meso-ITO|PSII with Q_B mimics carried out under monochromatic red light irradiation (10 mW cm^{-2}; λ = 685 nm): pyrroloquinoline quinone (methoxatin), 2,6-dichloro-1,4-benzoquinone (DCBQ), 2,6-ditert-butyl-1,4-benzoquinone (DTBpQ), and 3,5-ditert-butyl-1,2-benzoquinone (DTBoQ). The photocurrent of FTO|meso-ITO|PSII with DTBpQ was noticeably smaller than with DTBoQ, which was attributed to its limited aqueous solubility. DTBoQ was therefore selected as most suitable mediator for our PEC experiments. Conditions: 50 mM of MES buffer (pH 6.0) containing 50 mM KCl, 1 mM of Q_B mimics, N_2 atmosphere, room temperature, 0.25 cm^2 of geometrical surface area for all electrodes.

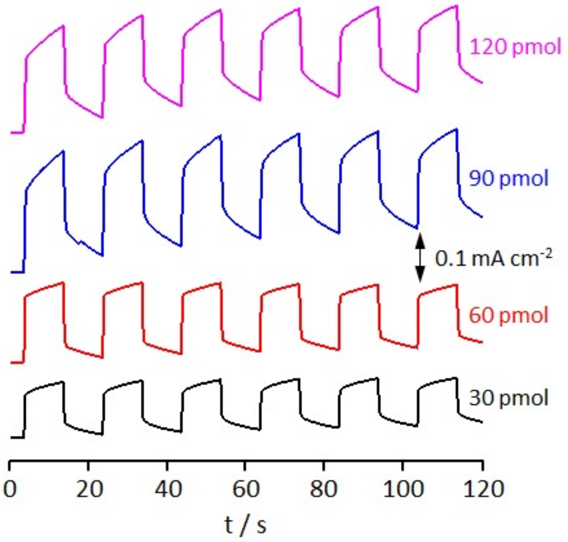

Figure S16. Controlled potential photoelectrolysis (CPPE) of FTO|IO-ITO|PSII with DTBoQ at 0.3 V *vs.* SHE (i.e., 0.663 V *vs.* RHE) under chopped light (100 mW cm^{-2}; AM1.5G; IR water filter; λ > 420 nm UV filter) to optimize PSII loading. Conditions: 50 mM of MES buffer (pH 6.0) containing 50 mM KCl, 1 mM of DTBoQ, N_2 atmosphere, room temperature, 0.5 cm^2 of geometrical surface area for all electrodes.

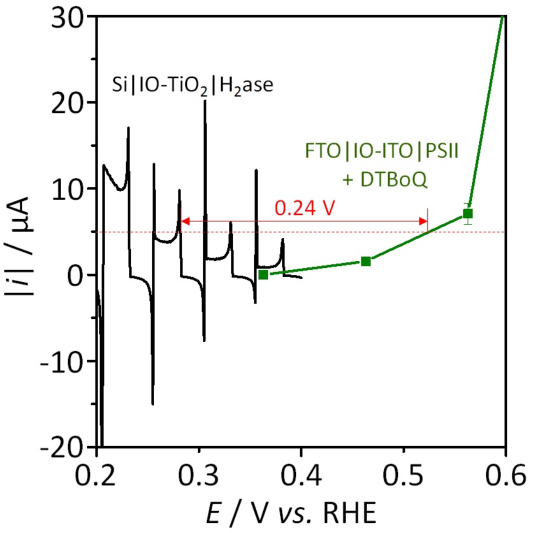

Figure S17. Overlay of a stepped chronoamperometry scan of FTO|IO-ITO|PSII with DTBoQ (green trace) and a chopped LSV scan of Si|IO-TiO_2|H_2ase (black trace) carried out in a three-electrode configuration under chopped simulated solar light (100 mW cm^{-2}; AM1.5G; IR water filter; $\lambda > 420$ nm). The current for Si|IO-TiO_2|H_2ase was inverted for ease of comparison. Conditions: 90 pmol of PSII, 80 pmol of H_2ase, 50 mM of MES buffer (pH 6.0) containing 50 mM KCl, 1 mM of Q_B mimics, geometrical surface areas (FTO|IO-ITO|PSII: 0.5 cm^2, Si|IO-TiO_2|H_2ase: 0.178 cm^2), N_2 atmosphere, room temperature.

Supporting References

[1] A. Volbeda, P. Amara, M. Iannello, A. L. De Lacey, C. Cavazza, J. C. Fontecilla-Camps, *Chem. Commun.* **2013**, *49*, 7061-7063.

[2] H. Kuhl, J. Kruip, A. Seidler, A. Krieger-Liszkay, M. Bünker, D. Bald, A. J. Scheidig and M. Rögner, *J. Biol. Chem.* **2000**, *275*, 20652–20659.

[3] A. I. Aria, K. Nakanishi, L. Xiao, P. Braeuninger-Weimer, A. A. Sagade, J. A. Alexander-Webber, S. Hofmann, *ACS Appl. Mater. Interfaces.* **2016**, *8*, 30564-30575.

[4] D. Mersch, C.-Y. Lee, J. Z. Zhang, K. Brinkert, J. C. Fontecilla-Camps, A. W. Rutherford, E. Reisner, *J. Am. Chem. Soc.* **2015**, *137*, 8541-8549.

[5] a) T. W. Kim, K.-S. Choi, *Science* **2014**, *343*, 990-994; b) Y.-H. Lai, D. W. Palm, E. Reisner, *Adv. Energy Mater.* **2015**, *5*, 1501668; c) Y. Kuang, Q. Jia, H. Nishiyama, T. Yamada, A. Kudo, K. Domen, *Adv. Energy Mater*. **2016**, *6*, 1501645.

[6] Y.-H. Lai, C.-Y. Lin, Y. Lv, T. C. King, A. Steiner, N. M. Muresan, L. Gan, D. S. Wright, E. Reisner, *Chem. Commun.* **2013**, *49*, 4331-4333.

[7] M. Kato, T. Cardona, A. W. Rutherford, E. Reisner, *J. Am. Chem. Soc.* **2012**, *134*, 8332−8335.

End of Supporting Information
